# Seasonal variation of immune response to heterologous erythrocytes in natural populations of red‐backed (*Clethrionomys rutilus*) and gray‐sided (*C. rufocanus*) voles in Western Siberia

**DOI:** 10.1002/ece3.9178

**Published:** 2022-08-04

**Authors:** Larisa B. Kravchenko, Konstantin A. Rogovin

**Affiliations:** ^1^ Tomsk State University Tomsk Russian Federation; ^2^ A.N. Severtsov Institute of Ecology and Evolution RAS Moscow Russian Federation

**Keywords:** antibody‐producing cells, *Clethrionomys rufocanus*, *Clethrionomys rutilus*, growth, immunocompetence, maturation

## Abstract

We studied the seasonal variation of adaptive humoral immunity (AHI) in northern red‐backed vole (*Clethrionomys rutilus* Pallas, 1779, RBV) and gray‐sided vole (*C. rufocanus* Sundevall, 1846, GSV) in Tomsk region of Western Siberia. Immunoresponsiveness (IR) to sheep red blood cells was assessed by the number of antibody‐producing cells in the spleen. The use of a generalized linear model to analyze the effects of species, sex, year of research, and season of withdrawal of individuals from nature on IR showed a significant effect of species identity, season of animal capture, and the interaction of species with season. The RBV demonstrated higher immune responses during a year, and both species had higher IR in winter. Suppression of IR in spring was greater, started earlier, and lasted longer (March–May) in GSV. In RBV, immunosuppression was restricted to April. The significant negative within year correlations of IR with body mass and masses of reproductive organs in GSV indicated a trade‐off between AHI and growth and reproduction processes. A probable explanation for the difference between species in the seasonal variation of AHI may be related to the difference in tropho‐energetic requirements of each vole species. GSV is a predominantly herbivorous rodent and its thermoregulation seems less efficient than of RBV. The deeper spring immunosuppression in GSV may explain in part its higher mortality during the season of colds.

## Graphical abstract

We studied seasonal variability of adaptive humoral immunity in the red‐backed vole (*Clethrionomys rutilus*) and the grey‐sided vole (*C. rufocanus*) in Western Siberia. Both species showed an increase in immunoreactivity in winter and immunosuppression, which differed in duration between the two species, in spring. We discuss possible reasons for the observed seasonal pattern of immunoreactivity changes and its species‐specific features.

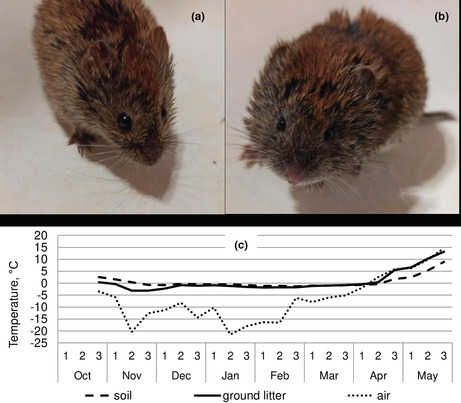

## 1 INTRODUCTION

Seasonal variation of immune functions has often been described in vertebrates (Martin et al., 2008), including humans (Dopico et al., 2015; Paynter et al., 2014). According to the winter immunoenhancement hypothesis (Sinclair & Lochmiller, 2000), immune responses among endothermic vertebrates of temperate zones should increase in winter due to the action of evolutionary‐determined endogenous bolstering mechanisms (Nelson, 2004; Nelson & Demas, 1996; Sinclair & Lochmiller, 2000) or from the winter decay of the trade‐off between reproduction and energetically costly immune functions (Greenman et al., 2005; Martin et al., 2004, 2006). Another possibility of winter enhancement of immunity involves changes in the abundance and distribution of pathogens and parasites over time (Gavier‐Widén & Mörner, 1993; Roth et al., 2018). Since contact‐transmitted diseases are more common during fall and winter in temperate regions of the world (Nelson, 2004), the enhancement of immune defenses may represent an effort to resist seasonal infections. Differences in life history strategies may also explain immunity enhancement in winter in some species and its absence in others (Lee, 2006; Martin et al., 2008; Nelson, 2004). Another problem is the limited opportunity to record the pattern of winter immunity enhancement in wild populations. The apparent consequences are that the underlying mechanisms observed in nature may overlap, can manifest partly, or may be completely indistinguishable, making it difficult or impossible to explain causality without special experiments. Indeed, the existing evidence for a winter enhancement of immunity based on observations is extremely controversial (Lohmiller & Moshkin, 1999; Martin et al., 2008). The phylogenetic mechanism that boosts the endogenous immune response may be disguised because winter stressors (low temperatures and food shortage) suppress the immune function. The cessation of reproduction in winter among seasonal breeders may lead to increased immune function through a reduced trade‐off between reproduction and immunity, but this may not become apparent because the action of winter stressors could have an immunosuppressive effect (Martin et al., 2008). Other reasons are also possible. In desert hamsters (*Phodopus roborovskii*), for instance, the lack of enhancement of adaptive humoral immunity in winter can be explained by higher winter energy metabolism (both basal and maximum metabolic rates), which is also associated with higher production of glucocorticoids (Vasilieva et al., 2020).

Despite the above limitations and negative notions about the low heuristic value of descriptive studies in seasonal variation of immune activity in nature (Martin et al., 2008), a comparison of phylogenetically close species that inhabit the same environment, but differ in certain eco‐physiological characteristics, might be valuable. We, therefore, analyzed two species of forest voles from similar habitats in Western Siberia, a region with a severe, strictly seasonal, and sharply continental climate.

**FIGURE 1:**
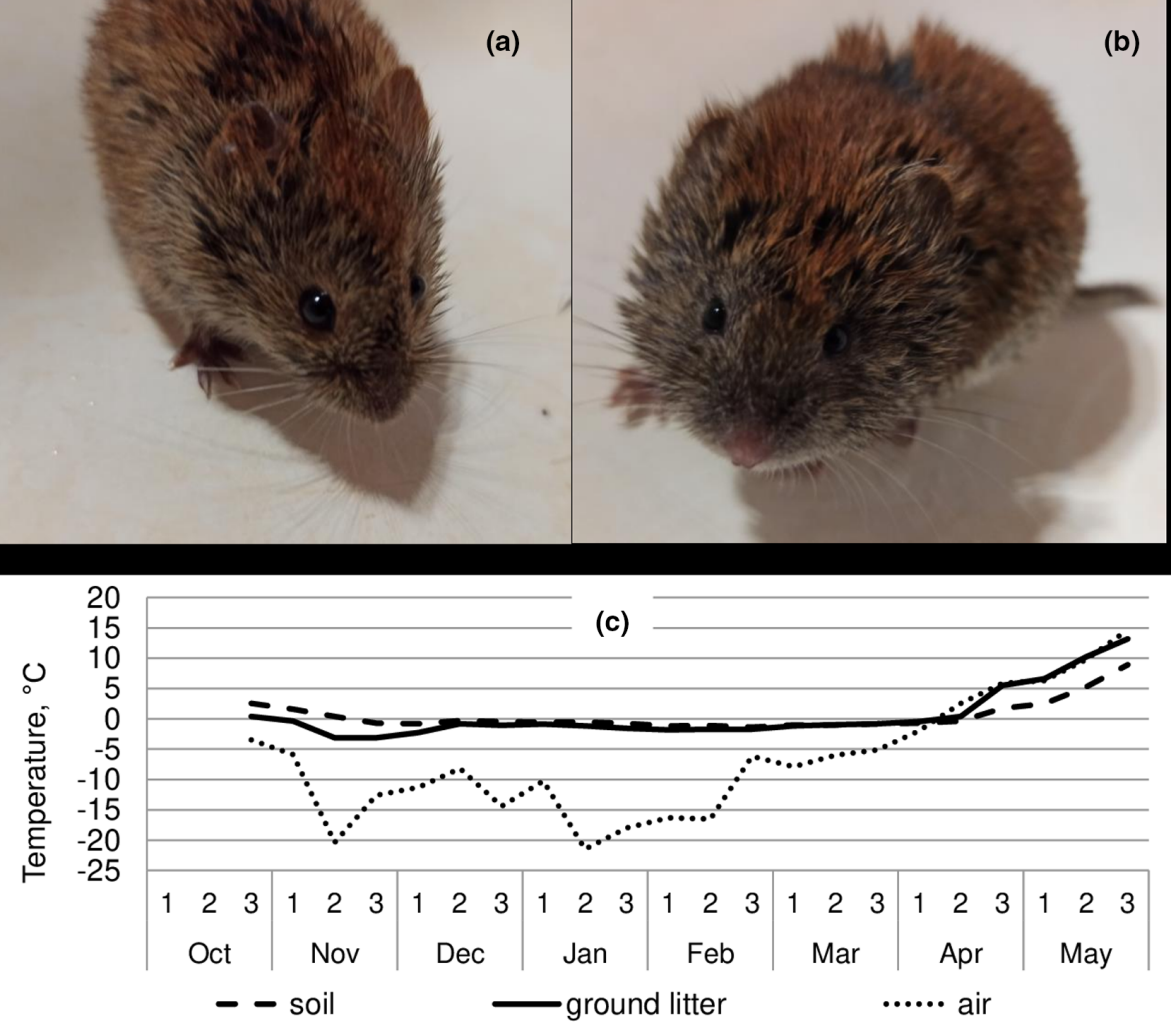
The studied species: (a) – red‐backed, (b) – gray‐sided vole. (c) – seasonal temperature variation. Measurements were conducted in the open air, in ground litter, and in soil at 15 cm depth using autonomous temperature recorders (DS1921G‐F5, Maxim Integrated Products, USA) every 3 h. The data are averages for every 10 days across 2 years of the registrations.

We report the results of our study of seasonal variation of adaptive humoral immunity in wintering generations of northern red‐backed vole (further in the text – red‐backed vole, *Clethrionomys rutilus* Pallas, 1779) and gray‐sided vole (*C*. (*Craseomys*) *rufocanus* Sundevall, 1846, (Figure 1))^1^ in the Tomsk region of the Russian Federation (N 56.475633°, E 85.128855°).

Both species overlap much spatially, occur within the same habitats, and demonstrate interspecific overlap in home ranges. They are active under the snow throughout the winter, even at air temperatures of −30° to −40°С, are generally similar in demography and population dynamic (Kravchenko, 1999), and experience a similar load of parasites and infections (Abramov et al., 2011; Krivopalov, 2011; Galbreath et al., 2013). At the same time, red‐backed and gray‐sided voles differ in their dietary habits and energetic performances. As a result, they behave differently in the winter. The red‐backed vole is much more granivorous, while the gray‐backed vole feeds mainly on vegetative parts of plants (Hansson, 1985; Koshkina, 1957; Soininen et al., 2013). At an ambient temperature of 5°С, the red‐backed vole demonstrates a higher metabolic rate (144 ml/g/min × 1000) and heat production (42 kcal/kg/h) vs 114 and 34, respectively, in the gray‐sided vole (Bashenina, 1977). Less developed mechanisms of heat production and, accordingly, less developed chemical thermoregulation in gray‐sided voles (Safronov, 2009) cause the behavioral adaptation for maintaining temperature homeostasis. Gray‐sided voles form wintering groups consisting of close relatives, mostly siblings (Ishibashi et al., 1998). In contrast, 60–70% of individuals of red‐backed vole overwinter individually (L. B. Kravchenko, *unpublished data*). The delayed dispersal of juveniles for successful wintering is associated in the gray‐sided vole with 1–1.5 months earlier cessation of maturation of underyearlings compared with the red‐backed vole (Kravchenko et al., 2012). Based on the above, we assumed that tropho‐energetic differences between the two species can affect the seasonal dynamics of immunocompetence, specifically, the energy‐costly system of adaptive humoral immunity (Buttgereit et al., 2000; Ots et al., 2001; Shudo & Iwasa, 2001) and could explain some of the demographic differences, in particular the high cold season mortality of gray‐sided voles (Hansen et al., 1999; Kusumoto & Saitoh, 2008; Saitoh et al., 2003). During the 3 years of our study, the relative abundance from fall to spring of the red‐backed vole varied from 1.1 to 1.8 times compared with 2.3 to 3.6 times in the gray‐sided vole (L. B. Kravchenko, *unpublished data*). We hypothesized that the earlier cessation of maturation in the gray‐sided vole compared with the red‐backed vole (Kravchenko et al., 2012) and the high mortality of the gray‐sided vole during seasons of colds can be related to characteristics of the seasonal dynamics of adaptive humoral immunity. We also examine whether the voles of each species exhibit a pattern of increased humoral immunoresponsiveness to an antigenic challenge in the harsh winter climate of Western Siberia, whether there are differences between species, and, if so, whether these differences can be associated with the species‐specific patterns of physiology and behavior.

Both species of forest voles in Siberia are typical “ephemerals” whose reproductive life is limited to one breeding season. Both species are characterized by two seasonal functional groups of individuals with different ontogenetic trajectories (Olenev, 2002), also named “spring and fall cohorts” (Gliwicz, 1996; Gliwicz et al., 1968; Zejda, 1971).

The first functional group consists of early born, fast maturing, and early dying young of the year, while the second group includes later born, wintering individuals that start breeding only in the next year. Since most individuals of the short‐lived spring cohort of voles in Western Siberia die in the fall (none of them survive the winter), we studied the seasonal variation of adaptive humoral immunoresponsiveness to sheep red blood cells (SRBC) in voles of the long‐lived wintering cohort. In red‐backed voles, most of the animals of this functional group are born in August. In the gray‐sided vole, the mass appearance of individuals of the wintering cohort occurs in July (Kravchenko et al., 2012). In September 2016–2021, the proportion of this functional group in our populations was 71–88%% in the red‐backed vole, and 79–92%% in gray‐sided vole. At the age of about 2 months old, growth slows down, maturation becomes delayed, and resumes only in March of the following year. Pregnant females appear in April (pregnancy lasts 18–19 days), their offspring are born in May, and leave the nests at 20 days old (late May–June). Females produce 2–3 litters, and in July, most of them die (among the trapped voles of each species in July 2016–2021, the proportion of overwintered animals varied from 0 to 16%% in red‐backed voles, and from 0 to 15%% in gray‐sided voles).

## 2 MATERIAL AND METHODS

### 2.1 Animals: habitat type, capture, keeping, sample sizes

We studied 94 red‐backed and 65 gray‐sided voles of the long‐lived wintering cohort. We caught voles to assess immunoresponsiveness from September to June in 2016–2017 and in 2017–2018 within one habitat type (mixed coniferous – parvifoliate forest: upper layer: *Picea obovata + Betula pendula + Abies sibirica + Populus tremula + Pinus sibirica*; middle layer: *Prunus padus + Sorbus aucuparia sibirica*; undergrowth: *Ribes rubrum + Ribes nigrum + Rosa majalis. + Rubus idaeus*). Temperature conditions at the voles' trapping site (*t C* ^o^) averaged for every 10 days during 2016–2017 and 2017–2018 are represented in Figure 1. The temperatures were measured from October to May in the open air, in the litter, and in the soil at a depth of 15 cm every 3 hours by autonomous recorders DS1921G‐F5 (Maxim Integrated Products, USA). Data on temperature were provided by S.I. Gashkov.

**FIGURE 2:**
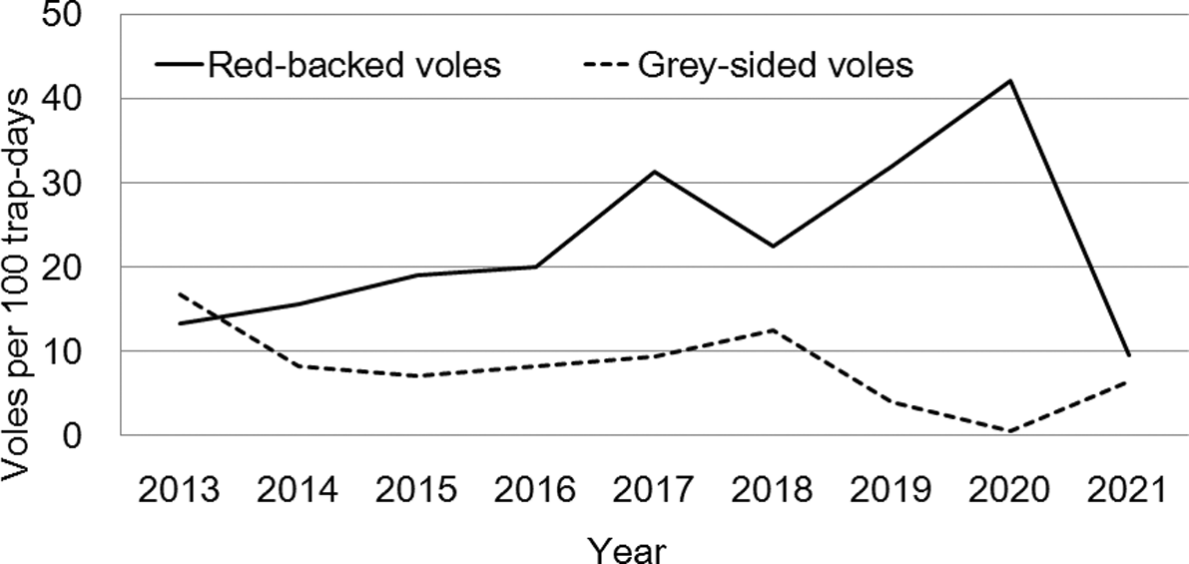
Long‐term dynamics of the relative abundance of the populations of red‐backed and gray‐sided voles according to data for July 2013–2021.

During the period of our study of immunoresponsiveness (2016–2018), the relative abundance (N individuals per 100 trap‐days) of the red‐backed vole continued to increase in 2016, the growth stopped in 2017, and in 2018, there was a slight decrease in the number. In the gray‐sided vole, a slow increase in the relative abundance occurred in 2016–2017; the growth stopped in 2018 (Figure 2). In the period 2016–2018, both populations demonstrated abundances close to long‐term averages.

From October to April, we caught voles at days with an air temperature not below −10°C. One to two days before catching, the live traps made of wire cloth (8 × 8 × 12) and not tightly covered with polyethylene film were placed into natural under‐snow holes visited by voles, under fallen trees, and branches. We identified the presence of voles by their feces. Distance between traps varied from 5 to 10 m. Folded toilet paper (1 m) was placed in the trap as nesting material. Surplus supply of bait consisted of sunflower seeds and carrots. On the day of capture, we opened the traps at 16:00 and examined them at 18:00 and 20:00. In case of capture, the vole, together with the trap, was immediately placed in a foam box (an ordinary thermal box) with plastic bags inside filled with warm (40–45°C) water (10 × 15 × 2 cm). Boxes with traps were transported to animal quarters in the lab. From May to September, we caught voles by live traps in lines with a distance of 5 m between traps. The traps were checked twice a day at 5–6 a.m. and at 7–8 p.m.

Animals we removed from nature were kept individually in cages (25 × 40 × 12 cm) under the natural photoperiod at a temperature of +8–+10°С from October to April, and at natural ambient temperatures for the remainder of the study. Food (oats, apples, grass), water, and nesting material were provided ad libitum.

We weighed the animals immediately after capture within an accuracy of 0.1 g and estimated the masses of the reproductive organs, the testes in males, and the uterus with ovaries in females, with an accuracy of 0.001 g after killing no later than 1 week after capture. To assess the between‐month variation of the masses of reproductive organs in order to increase the sample size, we used data for a longer period: for red‐backed vole from 2016 to 2018 (82 males and 59 females), for gray‐sided vole from 2016 to 2021 (93 males and 80 females).

### 2.2 Measurement of adaptive humoral immunoresponsiveness

We immunized voles from 8 to 9 am 36 h after capture. Tests of the duration of the glucocorticoid response to manipulations similar to trapping disturbance and to injection of ACTH in a congeneric species, *Clethrionomys glareolus*, showed that the effect of the stress factors ends within a day (Rogovin & Naidenko, 2010; Zavjalov et al., 2003). We did not treat pregnant females. To assess adaptive humoral immunity, we used local hemolysis in a liquid medium (Cunningham, 1965). We estimated the number of antibody‐producing cells (APC) of the spleen that were formed in response to the introduction of a non‐replicating antigen, 0.5 ml of 2% of SRBC suspension injected intraperitoneally. On the 5th day, the voles were killed by cervical dislocation. The suspension of spleen cells was prepared as described by Moshkin et al. (1998). The reaction mixture consisted of 500 μl spleen cell suspension, 500 μl washed SRBC (4 × 10^9^, erythrocytes/ml), and 500 μl lyophilized guinea pig serum (Biomed, Perm, Russia) resolved with 1 ml of isotonic sodium chloride solution. Cunningham chambers were prepared from glass microscope slides, loaded with 200 μl of reaction mixture (two chambers per individual), and incubated for 2 h at 37°C before hemolysis zones were counted. We calculated and then averaged the number of hemolysis zones (antibodies‐producing splenocytes) for each individual. To eliminate within and significant between species differences in body masses, the total number of APCs in the spleen was divided by individual body mass (Novikov et al., 2010). Estimation of the number of APCs per unit of body mass has an advantage over the estimate per unit of spleen mass, since splenomegaly is often observed in forest voles (10.6% in red‐backed vole and 7.7% in gray‐sided vole in our study). An increase in the mass of the spleen in such cases is not associated with an increase in the number of APCs (Kravchenko, *unpublished*). The number of APCs per unit of body mass was used as the main indicator of immune activity. To analyze correlations of immunoresponsiveness with body mass and with masses of reproductive organs, we used the absolute number of APCs.

### 2.3 Statistical procedures

All statistical analyses were performed using STATISTICA v. 10.0 (StatSoft Inc., USA). To evaluate the effects of a set of independent variables on the magnitude of humoral immunoresponsiveness to SRBC (the number of antibody‐producing splenocytes per unit body mass), we applied Generalized Linear Model (GLZ) analysis with the normal condition and identity link function. The intensity of humoral immune response (APCs/body mass) was assigned as a dependent variable. Its values were log‐transformed (ln) preliminary to normalize the distribution. Predictors included four categorical variables (species, sex, year of research, and season of withdrawal of voles with four gradations: winter, spring, summer, and fall). Two‐way interactions between predictors were also considered. The significance of predictors and their interactions was tested using Wald statistics and Likelihood ratio test type III.

For a more detailed study of within year variation of the immunocompetence of each of the vole species, we studied the monthly variation of immunoresponsiveness to SRBC. The monthly variation of body mass and mass of reproductive organs (testes in males, and uterus + ovary in females) was also considered. We used Kolmogorov–Smirnov test and visual examination of histograms to examine distributions. Levene's test was used to check homogeneity of variances within groups. Since the assumption of normality and homogeneity of variances have not been rigorously confirmed for the seasonal variation of immunoresponsiveness and were notably unconfirmed for monthly variation of immunoresponsiveness, body mass, and mass of reproductive organs, to compare differences between seasons and months, we used Kruskal–Wallis ANOVA with multiple (post hoc) comparisons of average ranks for each pair of groups (normal *z*‐values were computed for each comparison, as well as post hoc probabilities corrected for the number of comparisons for a two‐sided test of significance; Siegel & Castellan, 1988). We also used Mann–Whitney U test for independent pair comparisons. Spearman's Rank‐Order Correlation Coefficient (Rs) was used to measure linkage of continuous non‐normally distributed data. Since our analyses were based in part on data from a different number of years (we analyzed the between‐month variation in the mass of the reproductive organs of voles over a longer time interval, than immunoresponsiveness), we indicated the sample sizes in the headings to the table and figures. Tests were two‐sided, with a significance level < 0.05.

### 2.4 Compliance with regulations when working with animals

We conducted all procedures involving the experimental animals in accordance with the requirements of the Bioethical Commission of the Biological Institute at Tomsk State University, Russia (approval no 40 of November 1, 2021), and in compliance with laws and regulations of Russia, Association for the Study of Animal Behaviour/Animal Behavior Society Guidelines for the Use of Animals in Research (Animal Behaviour, 2018, 135, I‐X), and the European Convention for the Protection of Vertebrate Animals Used for Experimental and Other Scientific Purposes (ETS No. 123 of March 18, 1986). Every effort was made to minimize the number of voles used and their discomfort and suffering.

## 3 RESULTS

### 3.1 Factors affecting immunoresponsiveness to SRBC

The use of GLZ to analyze the effects of species, sex, year of research, and season of withdrawal of individuals from nature on spleen immunoresponsiveness to antigenic challenge showed significant effects of species identity and season of animal capture. The only one interaction of predictors with the probability close to the significant level was the interaction of species with season (Table 1).

**TABLE 1:** Effects of species, sex, year, season of withdrawal (winter, spring, summer end fall) and interaction of species with season on the adaptive humoral immunity response to SRBC in red‐backed vole and grey‐sided vole. *B* and *SE* correspond to parameter estimates and standard errors in GLZ with normal distribution and identity link function; *W* corresponds to Wald statistic estimates and *X* ^2^ corresponds to likelihood ratio test type III. Significant effects (*p* < .05) are marked in bold. N = 159.

Model and predictors	Level of effect	Response and statistics
Species	1	***B* = 0.704 ± 0.090**, df = 1, ***χ*** ^**2**^ **= 51.331, *p* < .001**
Sex	1	*B* = 0.092 ± 0.083, df = 1, *χ* ^2^ = 1.25, *p* = .264
Year	1	*B* = −0.017 ± 0.081, df = 1, *χ* ^2^ = 0.046, *p* = .83
Season of withdrawal		df = 3, ***χ*** ^**2**^ **= 70.076**, ***p* < .001**
	1	***B* = 1.150 ± 0.144, *W* = 63.751, *p* < .001**
	2	***B* = −0.706 ± 0.129, *W* = 29.825, *p* < .001**
	3	***B* = −0.557 ± 0.199, *W* = 7.797, *p* = .052**
Species*Season of withdrawal		df *=* 3, *χ* ^2^ = 7.545, *p* = .056
	1	*B* = −0.082 ± 0.143, *W* = 0.327, *p* = .567
	2	***B* = 0.299 ± 0.126, *W* = 5.629, *p* = .018**
	3	*B* = 0.025 ± 0.199, *W* = 0.016, *p* = .899

### 3.2 Seasonal variation in immune responsiveness to antigenic challenge

**FIGURE 3:**
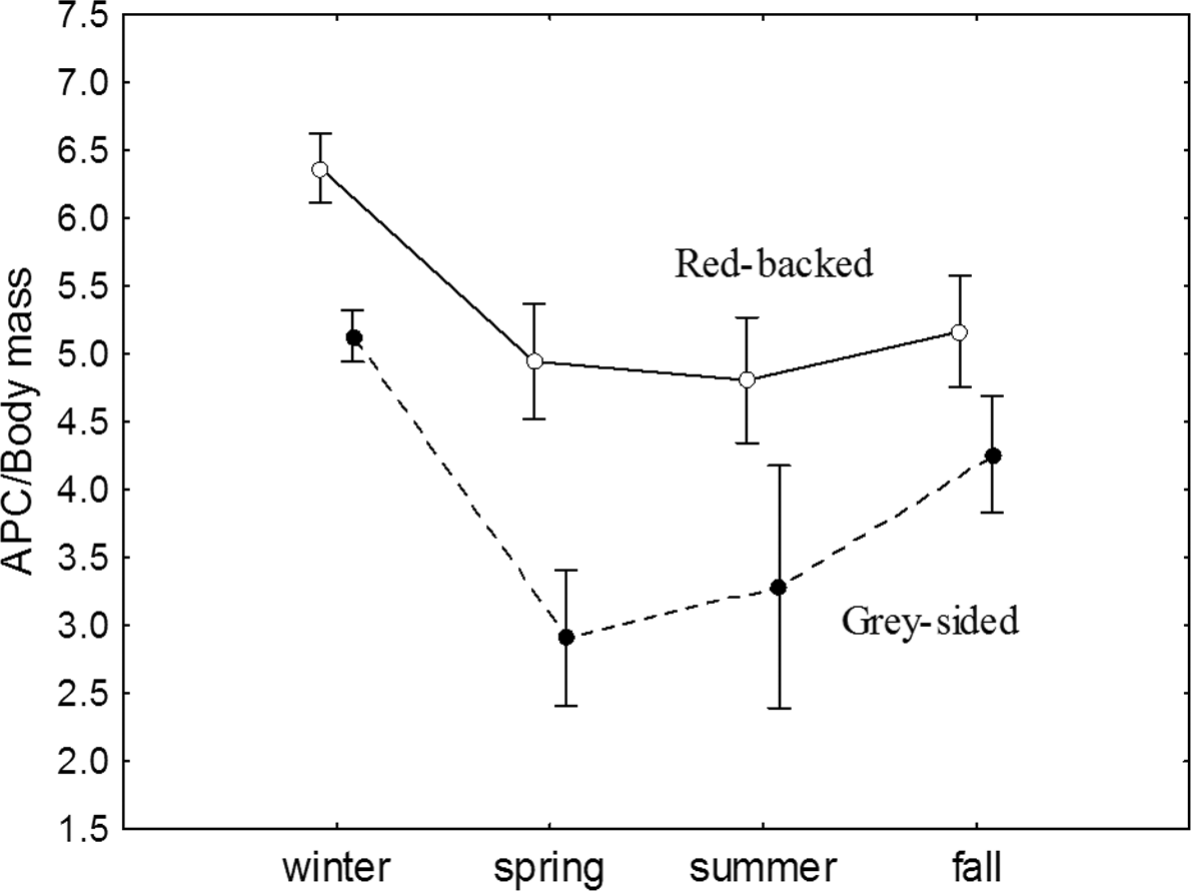
Seasonal fluctuations of immunoresponsiveness (the number of antibody‐producing cells (APC) per body mass unite, Ln) in response to injection of SRBC in the wintering generations of red‐backed (*N* = 94) and gray‐sided (*N* = 65) voles. Weighted marginal means with 0.95% confidence intervals are shown (GLZ: Species*season: Wald *χ* ^2^ = 7.73, *p* = .05).

**FIGURE 4:**
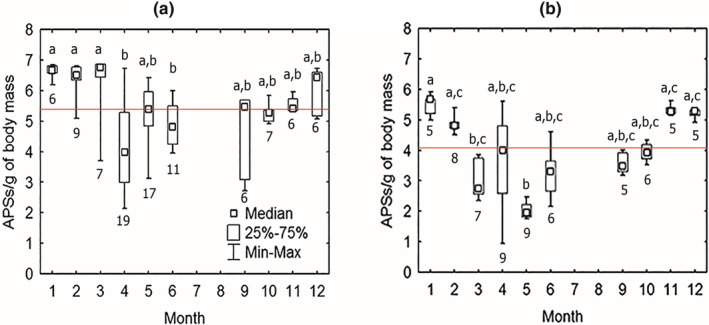
Between‐month variation of immunoresponsiveness (the number of antibody‐producing cells (APC) per body mass unite, Ln) in response to injection of SRBC in the wintering generations of (a) red‐backed (*N* = 94) and (b) gray‐sided (*N* = 65) voles. Kruskal–Wallis ANOVA for red‐backed vole: *H* (9, *N* = 94) = 46.43, *p* < .001, for gray‐sided vole: *H* (9, *N* = 65) = 49.07, *p* < .001. Square dots, boxes, and whiskers denote medians and limits of variation. Identical letters indicate absence of significant differences in post hoc multiple comparisons in Kruskal–Wallis ANOVA (*p* < .05). Numbers below the box plot of the corresponding month indicate sample sizes. Solid line designates within year median value of immunoresponsiveness.

The red‐backed vole demonstrated higher immunoresponsiveness to SRBS compared with the gray‐sided vole during a year (Mann–Whitney *U* Test: *Z* = 6.24, *N*_red‐backed_ = 94, *N*_grey‐sided_ = 65, *p* < .001; Figure 3.) Analysis of seasonal effects on immunoresponsiveness to antigenic challenge in each species (red‐backed vole: Kruskal–Wallis test: *H* [3, *N* = 94] = 5.59, *p* < .001; gray‐sided vole: Kruskal–Wallis test: *H* [3, *N* = 65] = 33.22, *p* < .001) showed that red‐backed voles had a higher immune response to SRBC in the winter compared to other seasons (post hoc multiple comparisons: winter–spring: *Z* = 4.45.19, *p* < .001; summer‐winter: *Z* = 4.10, *p* < .001; fall–winter: *Z* = 3.57, *p* < .01). Gray‐sided vole also demonstrated significantly higher immune response in winter compared with summer (*Z* = 3.22, *p* < .01), but not with the fall season (*Z* = 2.17, *p* = .20). Gray‐sided voles had deeper spring suppression of the immune response compared with winter (*Z* = 5.52, *p* < .001), and the immunoresponsiveness tended to increase from spring through summer to fall (spring‐fall difference: *Z* = −3.05, *p* < .05). The highest difference between species in immunoresponsiveness was also pronounced in the spring (Mann–Whitney *U* Test: *Z* = 4.99, *N*_*C.rutilus*_ = 43, *N*_*C.rufocanus*_ = 25, *p* < .001). Regarding differences between months, we found that gray‐sided voles showed an earlier onset of spring immunosuppression that started in March and continued to May reaching its lowest median values in May (Figure 4b). In the red‐backed vole, there was no difference between winter months and March, and the lowest median value was in April (Figure 4a).

### 3.3 Growth, maturation, and immunocompetence

**FIGURE 5:**
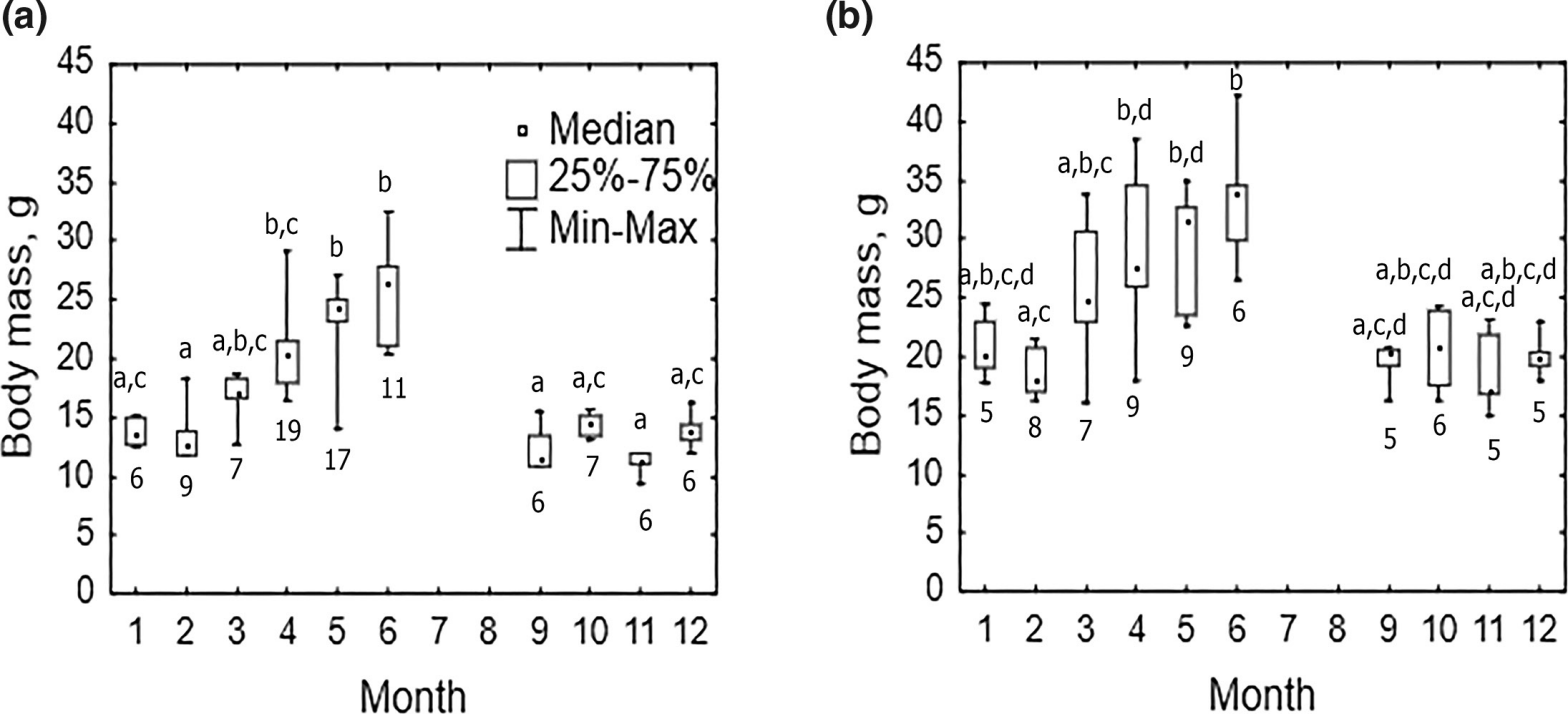
Between‐month variation of body mass in wintering (a) red‐backed vole (Kruskal–Wallis ANOVA: *H* (9, *N* = 94) = 76.20 *p* < .001) and (b) gray‐sided vole (Kruskal–Wallis ANOVA: *H* (9, *N* = 65) = 38.03, *p* < .001). Square dots, boxes, and whiskers denote medians and limits of variation. Identical letters indicate absence of significant differences in post hoc multiple comparisons in Kruskal–Wallis ANOVA (*p* < .05). Numbers below the box plot of the corresponding month indicate sample sizes.

**FIGURE 6:**
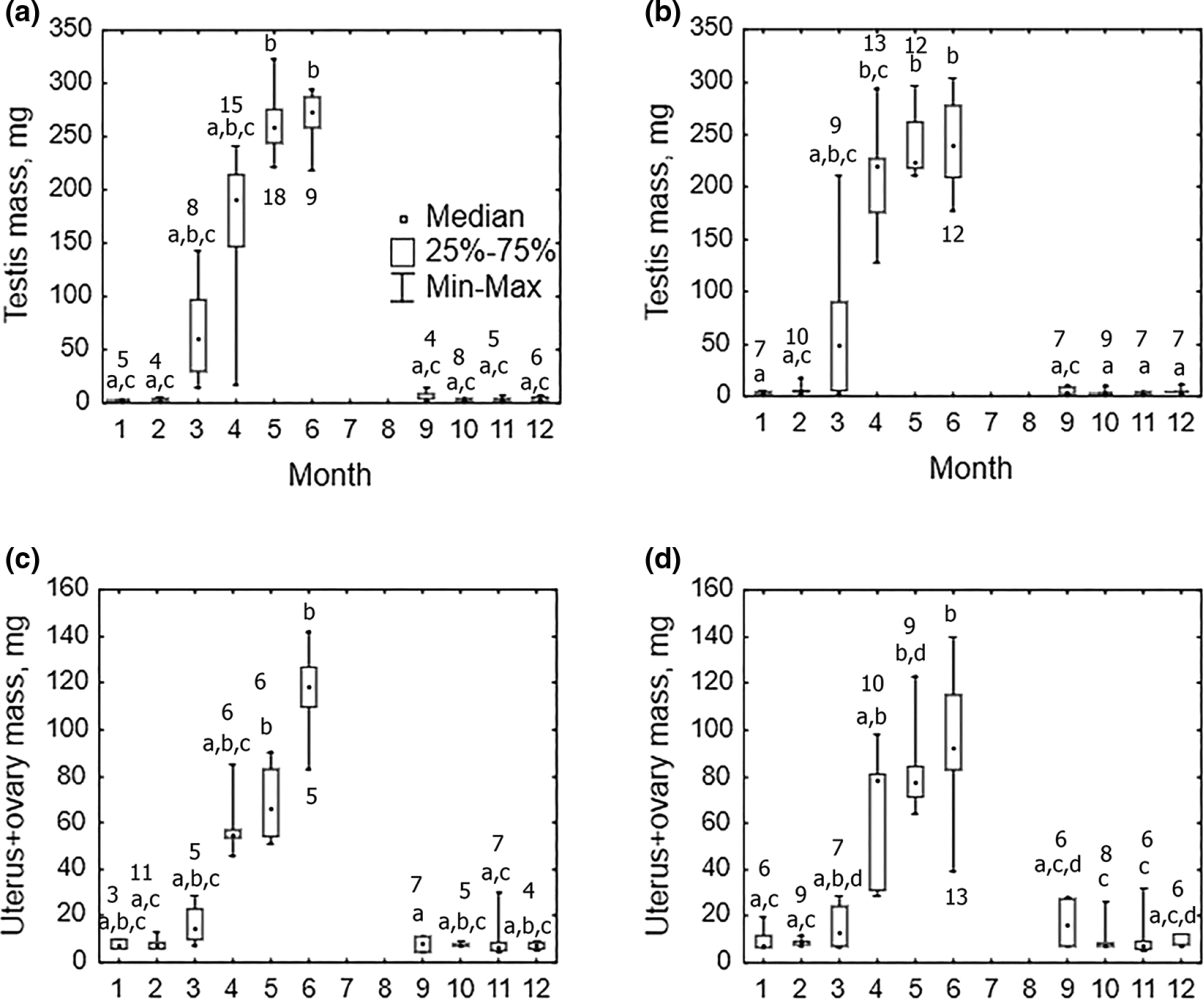
Between‐month variation of testis mass (means for pair, mg) in wintering (a) red‐backed (*N* = 82) and (b) gray‐sided (*N* = 93) voles, and between‐month variation of uterus + ovary mass (mg) in wintering (c) red‐backed (*N* = 59) and (d) gray‐sided (*N* = 80) voles. Square dots, boxes, and whiskers denote medians and limits of variation. Identical letters indicate absence of significant differences in post hoc multiple comparisons in Kruskal–Wallis ANOVA (*p* < .05). Numbers near the box plot of the corresponding month indicate sample sizes. Kruskal–Wallis test: red‐backed vole: testes mass: *H* (9, *N* = 82) = 72.69, *p* < .001, uterus + ovary mass: *H* (9, *N* = 59) = 40.07, *p* < .001; gray‐sided vole: testes mass: *H* (9, *N* = 93) = 72.56, *p* < .001; uterus + ovary mass: *H* (9, *N* = 80) = 59.79, *p* < .001.

Both species of voles of wintering generation stopped growth in September and survived the winter with unchanged body mass and with immature reproductive system. The growth of body masses and masses of reproductive organs started in both species in March and continued in a similar manner (Figures 5 and 6). In the red‐backed vole, the immunoresponsiveness, assessed by the absolute number of antibody‐producing cells (APC), did not depend on body mass and masses of reproductive organs within a year, although the signs of Spearman's rank correlation coefficients were negative in all cases. In the gray‐sided vole, Spearman's Rs were negative and higher, and statistically significant in females (Table 2).

**TABLE 2:** Spearman rank correlations of immunoresponsiveness to SRBC (total number of antibody‐producing cells, *N* APC) with body mass and masses of reproductive organs in red‐backed and grey‐sided voles within a year (2016‐2018). Significant effects (*p* < .05) are marked in bold. N=159.

Species	Predictors	Males			Females		
		Rsp	*n*	*p*	Rsp	*n*	*p*
*C. rutilus*	Body mass, g	−0.09	60	.484	−0.17	34	.325
	Testes mass, g	−0.12	60	.364			
	Uterus + ovary mass, g				−0.3	34	.088
*C.rufocanus*	Body mass, g	−0.25	35	.144	−0.39	30	**.033**
	Testes mass, g	−0.32	35	.059			
	Uterus + ovary mass, g				−0.6	30	**<.001**

## 4 DISCUSSION

Season affected the immunoresponse of forest voles caught in nature. We found a statistically significant effect on the magnitude of the immune response to SRBC in both vole species. Both species exhibited a greater immunoresponse in winter with the rather low variance in values. Species differed, however, in their immunoresponses at other times of the year. In the spring, the gray‐sided voles showed a suppressed immunoresponse that was deeper, began earlier, and lasted longer than in the red‐backed vole. Immunosuppression in the red‐backed vole was high but restricted to April.

The year of capture and sex of the voles seemed to have no effect on their immune response. Because the red‐backed and gray‐sided voles have promiscuous mating systems (Gromov, 2008), we hypothesized that there may be sex differences in their humoral immune responsiveness to an antigenic challenge (Zuk & Stoehr, 2002), but we were unable to confirm this.

Our results did partly agree with data from other studies that assessed the seasonal immunoresponsiveness to SRBC of bank (*C. glareolus*) and red‐backed voles (Moshkin et al., 1998). The magnitude of the humoral immune response to the antigenic challenge increased in these species of voles from summer to fall. In our study, the immune response in the red‐backed vole was higher throughout the year, and the difference in immunoresponsiveness between winter and other seasons was statistically significant; in the gray‐sided vole, the difference between winter and other seasons was also significant, but with the exception of fall.

The winter immunity enhancement hypothesis (Nelson, 2004; Nelson & Demas, 1996; Sinclair & Lochmiller, 2000) and the limitations to testing it have been critically discussed in the past (Martin et al., 2008). As an alternative to the evolutionarily determined endogenous mechanism of enhancing immune activity to counteract winter stressors, the trade‐off hypothesis between costs for reproduction and immune defense has been proposed (Greenman et al., 2005; Martin et al., 2004, 2006). These two explanations are not mutually exclusive, and it is impossible to give preference to one or another mechanism without special experiments (Martin et al., 2008). In our case study of two closely related species of forest voles, based on observations, we can only offer hypothetical explanations that seem to us the most plausible.

Traditionally, stress from low temperatures and limited availability of resources is considered to be a factor negatively affecting immunoresponsiveness in winter (Martin et al., 2008; Nelson, 2004). An increase in the level of corticosterone in September and October in both species of voles was previously revealed (Kravchenko et al., 2016). This could have a negative effect on the immune system during the preparation of physiological systems for winter. However, according to the results of the present study, this does not cause a seasonal decrease in humoral immunoresponsiveness in voles. This may be due to the relatively mild winters of the last two decades. In Western Siberia, climate warming manifested mainly in winter (Gordov et al., 2011). Winter's increasing mildness in areas with traditionally cold winters can reduce the risk of death and preserve the health of people and livestock (Lacetera, 2019). In rodents, milder winters may be the reason for the lack of winter immunosuppression which could be caused by the low temperature stress (Kusumoto & Saitoh, 2008; Xu & Hu, 2017). In the framework of the winter immunity enhancement hypothesis, it seems possible that the lower level of winter stress associated with the warming climate in Siberia (Gordov et al., 2011) makes the winter endogenous bolstering of immunoresponsiveness visible. Winter temperatures (10‐day averages) in 2016–2018 did not fall below −22°C and mostly fluctuated within −10° to −15°C. Increased immunocompetence in winter in both species of voles associated with lower variance also may be related to their young age and delayed growth and maturation during the fall–winter period. The resumption of growth and maturation processes begins in both species in March and proceeds in a similar way. The spring suppression of humoral immunoresponsiveness to antigenic challenge can be explained precisely by these costly processes which recommence against the background of low ambient temperatures and limited food availability. These external stressors may also provide an additional contribution to the limitation of immunoresponsiveness (Kusumoto & Saitoh, 2008).

Given the limitations in interpreting the causes of seasonal variation in immune activity in nature (Martin et al., 2008), we nevertheless made an attempt to explain the interspecific differences based on the physiology and behavior of these phylogenetically related species of forest voles. A deeper spring suppression of immunoresponsiveness and, accordingly, a wider seasonal variation of immunocompetence in the gray‐sided vole are consistent with within year antagonistic relationships between the magnitude of the immune response to the antigen, on the one hand, and the body and reproductive organs masses, on the other. The negative relationship, which was strongest in females of gray‐sided voles, supports a trade‐off between the processes of growth and maturation and the activity of the adaptive humoral immunity system. The activity of this immune system, according to some estimates, seems to be costly for the organism (Buttgereit et al., 2000; Ots et al., 2001; Shudo & Iwasa, 2001, but see Martin et al., 2008). In contrast to the red‐backed vole, the suppression of immunoresponsiveness in the gray‐sided vole in spring was not only greater but it also started earlier (March) and lasted longer (May). These differences, in our opinion, cannot be easily explained by a cardinal difference in the phase of the population cycle. In fact, during the period 2016–2018, vole populations showed a relative abundance close to the long‐term averages. Another possible explanation based solely on the difference in seasonal growth and maturation dynamics out of context of trophic and thermo‐energetic adaptations is also untenable. In our study, both species did not differ in growth and maturation parameters. In our opinion, the most probable explanation should be sought in the tropho‐energetic differences between the two vole species. Differences in the seasonal dynamics of immunoresponsiveness probably reflect the specificity of species in provision of the immune system with resources. The birth of litters by the predominantly granivorous red‐backed vole occurs as a rule from mid‐May. The mass birth of litters started in the gray‐sided vole, predominantly herbivorous rodent, a week later (L. B. Kravchenko, *unpublished data*). The immunosuppression in April in the red‐backed vole can be explained by continued growth and maturation, which turn into mating and reproduction against the backdrop of still low ambient temperatures and limited food availability. The recovery of the immune responsiveness in May indicates that the shortage in available resources has been overcome. At this time, red‐backed voles breed intensively, growth is completed, and the temperature of the environment appears to be quite comfortable for the species. In the gray‐sided vole, spring immunosuppression in March coincided with the beginning of growth and maturation of the animals. It is possible that in comparison with the red‐backed vole, the lower metabolic rate at low temperatures (Bashenina, 1977) in the predominantly herbivorous gray‐sided vole (Koshkina, 1957; Soininen et al., 2013) limits the scope for an intense humoral immune response to antigenic challenges. At the end of April, the wintering groups of relatives (Ishibashi et al., 1998) in the gray‐sided vole become disintegrated, and voles form individual home ranges. It has been shown that at 5°C with food provided ad libitum, individual caging of gray‐sided voles causes immunosuppression (Kusumoto & Saitoh, 2008). Although we observed a well pronounced between individual variation in immune responsiveness in April, the variation decreased again and the values became minimal in May. The possible reason for the pattern we observed in gray‐sided vole could be a combination of the effects of changing social structure and intensive reproductive effort against the background of still rather low ambient temperatures, not optimal for the species (Figure 1). It is possible that deep and prolonged spring immunosuppression in the gray‐sided vole may be one of the reasons for the high mortality during the cold season described for this species (Hansen et al., 1999; Kusumoto & Saitoh, 2008; Saitoh et al., 2003).

Thus, under the conditions of Western Siberia, the differences between two species of forest voles in the depth and duration of spring suppression of adaptive humoral immunity may result from the specifics of the mechanisms for maintaining temperature homeostasis. The lower level of metabolism and the less developed mechanism of chemical thermoregulation in the mostly herbivorous gray‐sided vole (Safronov, 2009), combined with the compensatory features of winter behavior, can explain the longer spring immunosuppression in this species.

## AUTHOR CONTRIBUTIONS

**Larisa B. Kravchenko:** Conceptualization (equal); data curation (equal); formal analysis (equal); funding acquisition (lead); investigation (equal); methodology (equal); project administration (equal); resources (equal); software (supporting); supervision (equal); validation (equal); visualization (equal); writing – original draft (lead); writing – review and editing (supporting). **Konstantin A. Rogovin:** Conceptualization (equal); data curation (equal); formal analysis (equal); funding acquisition (supporting); investigation (equal); methodology (equal); project administration (equal); resources (equal); software (lead); supervision (equal); validation (equal); visualization (equal); writing – original draft (supporting); writing – review and editing (lead).

## CONFLICT OF INTEREST

The authors declare that there is no conflict of interests.

## Data Availability

Dataset entitled, "Humoral immunity of voles Cricetidae Rodentia in Siberia". doi: 10.5061/dryad.dbrv15f43 URL: https://datadryad.org/stash/share/Mf2Gn‐bs5MY1Iwz1Ko2VJodiKpWQQj8noHLtEjyQu0Y.

## Endnote

^1^
We are following the recent recommendation to use the former genus name of the forest voles, *Clethrionomys* instead of *Myodes* (Kryštufek et al., 2020), and use it traditionally for both species, although according to some preliminary data, *C. rufocanus* can be classified phylogenetically as a separate genus *Craseomys* (Lebedev et al., 2007)

## References

[ece39178-bib-0001] Abramov, S. A. , Yashina, L. N. , Dupal, T. A. , Zdanovskaya, N. I. , Protopopova, E. V. , Pozdnyakov, A. A. , Krivopalov, A. V. , & Petrovsky, D. V. (2011). New data on the distribution of hantavirus in rodent populations in Siberia. Contemporary Problems of Ecology, 4(4), 410–415.

[ece39178-bib-0002] Bashenina, N. V. (1977). Adaptive specific features of heat exchange in mouse‐like rodents (p. 296). М.: MSU Publishing house.

[ece39178-bib-0003] Buttgereit, F. , Burmester, G.‐R. , & Brand, M. D. (2000). Bioenergetics of immune functions: Fundamental and therapeutic aspects. Immunology Today, 21(4), 194–199. 10.1016/s0167-5699(00)01593-0 10740243

[ece39178-bib-0004] Cunningham, A. J. (1965). A method of increased sensitivity for detecting single antibody‐forming cells. Nature, 207, 1106–1107. 5866316 10.1038/2071106a0

[ece39178-bib-0005] Dopico, X. C. , Evangelou, M. , Ferreira, R. C. , Guo, H. , Pekalski, M. L. , Smyth, D. J. , & Todd, J. A. (2015). Widespread seasonal gene expression reveals annual differences in human immunity and physiology. Nature Communications, 6(1), 1–13. 10.1038/ncomms8000 PMC4432600 25965853

[ece39178-bib-0006] Galbreath, K. E. , Ragaliauskaite, K. , Kontrimavichus, L. , Makarikov, A. A. , & Hoberg, E. P. (2013). A widespread distribution for *Arostrilepis tenuicirrosa* (Eucestoda: Hymenolepididae) in *Myodes* voles (Cricetidae: Arvicolinae) from the Palearctic based on molecular and morphological evidence: Historical and biogeographic implications. Acta Parasitologica, 58(4), 441–452. 24338304 10.2478/s11686-013-0170-6

[ece39178-bib-0007] Gavier‐Widén, D. , & Mörner, T. (1993). Descriptive epizootiological study of European brown hare syndrome in Sweden. Journal of Wildlife Diseases, 29(1), 15–20. 8383252 10.7589/0090-3558-29.1.15

[ece39178-bib-0008] Gliwicz, J. (1996). Life history of voles: Growth and maturation in seasonal cohorts of the root vole. Miscellania Zoologica, 19(1), 1–12.

[ece39178-bib-0009] Gliwicz, J. , Andrzejewski, R. , Bujalska, G. , & Petrusewicz, K. (1968). Priductivity investigation of an Island population of *Clethrionomys glareolus* (Schreber, 1780). I. Dynamics of cohorts. Acta Theriologica, 13, 401–413.

[ece39178-bib-0010] Gordov, E. P. , Bogomolov, V. Y. , Genina, E. Y. , & Shulgina, T. M. (2011). Analysis of regional climate processes in Siberia: Approach, data and some results. Bulletin of the Novosibirsk State University Series: Information Technologies, 9(1), 56–66.

[ece39178-bib-0011] Greenman, C. G. , Martin, L. B. , & Hau, M. (2005). Reproductive state, but not testosterone, reduces immune function in male house sparrows (*Passer domesticus*). Physiological and Biochemical Zoology, 78, 60–68. 10.1086/425194 15702464

[ece39178-bib-0012] Gromov, V. S. (2008). Spatial‐ethologic structure of rodent populations (p. 581). М.: KMK Scientific Press Ltd.

[ece39178-bib-0013] Hansen, T. F. , Stenseth, N. C. , & Henttonen, H. (1999). Multiannual vole cycles and population regulation during long winters: An analysis of seasonal density dependence. American Naturalist, 154, 129–139. 10.1086/303229 29578785

[ece39178-bib-0014] Hansson, L. (1985). *Clethrionomys* food: Generic, specific and regional characteristics. Annales Zoologici Fennici, 22, 315–318.

[ece39178-bib-0015] Ishibashi, Y. , Saitoh, T. , & Kawata, M. (1998). Social organization of the vole *Clethrionomys rufocanus* and its demographic and genetic consequences: A review. Researches on Population Ecology, 40(1), 39–50.

[ece39178-bib-0016] Koshkina, T. V. (1957). Comparative ecology of red‐backed voles in Northern taiga (pp. 3–65). М.: MSU Publ. House.

[ece39178-bib-0017] Kravchenko, L. B. (1999). Community dynamics and population specific features of woodland voles (р. *Clethrionomys*) in the Ob River bottomland. PhD Biology Thesis Research, 157 p.

[ece39178-bib-0018] Kravchenko, L. B. , Moskvitina, N. S. , & Zavyalov, E. L. (2016). Dynamics of the fecal corticosterone content in males of red, gray‐sided, and bank voles (*Myodes*, Rodentia, Cricetidae) upon sexual maturation. Biology Bulletin, 43(9), 45–54.

[ece39178-bib-0019] Kravchenko, L. B. , Zavjalov, E. L. , & Moskvitina, N. S. (2012). Sexual maturation and age‐related dynamics of corticosterone in *Clethrionomys rutilus* and *Cl. rufocanus* voles (Rodentia, Cricetidae) under experimental conditions. Biology Bulletin, 39(7), 627–633.

[ece39178-bib-0020] Krivopalov, A. V. (2011). Fauna and ecology of helminths of myomorpha murid rodents of the black taiga of the North‐Eastern Altai. PhD Biology Thesis Research, 148 p.

[ece39178-bib-0021] Kryštufek, B. , Tesakov, A. S. , Lebedev, V. S. , Bannikova, A. A. , Abramson, N. I. , & Shenbrot, G. (2020). Back to the future: The proper name for red‐backed voles is *Clethrionomys* Tilesius and not *Myodes* Pallas. Mammalia, 84(2), 214–217.

[ece39178-bib-0022] Kusumoto, K. , & Saitoh, T. (2008). Effects of cold stress on immune function in the grey‐sided vole, *Clethrionomys rufocanus* . Mammal Study, 33, 11–18.

[ece39178-bib-0023] Lacetera, N. (2019). Impact of climate change on animal health and welfare. Animal Frontiers, 9(1), 26–313. 32002236 10.1093/af/vfy030 PMC6951873

[ece39178-bib-0024] Lebedev, V. S. , Bannikova, A. A. , Tesakov, A. S. , & Abramson, N. I. (2007). Molecular phylogeny of the genus *Alticola* (Cricetidae, Rodentia) as inferred from the sequence of the cytochrome b gene. Zoologica Scripta, 36, 547–563.

[ece39178-bib-0025] Lee, K. A. (2006). Linking immune defenses and life history at the levels of the individual and the species. Integrative and Comparative Biology, 46(6), 1000–1015. 21672803 10.1093/icb/icl049

[ece39178-bib-0026] Lohmiller, R. L. , & Moshkin, M. P. (1999). The adaptive significance of the variability of immunocompetence in populations of small mammals. Siberian Ecological Journal, 1, 37–58.

[ece39178-bib-0027] Martin, L. B. , Hasselquist, D. , & Wikelski, M. (2006). Immune investments are linked to pace of life in house sparrows. Oecologia, 147, 565–575. 10.1007/s00442-005-0314-y 16450181

[ece39178-bib-0028] Martin, L. B. , Pless, M. , Svoboda, J. , & Wikelski, M. (2004). Immune activity in temperate and tropical house sparrows: A common‐garden experiment. Ecology, 85, 2323–2331. 10.1890/03-0365

[ece39178-bib-0029] Martin, L. B. , Weil, Z. M. , & Nelson, R. J. (2008). Seasonal changes in vertebrate immune activity: Mediation by physiological trade‐offs. Philosophical Transactions of the Royal Society B: Biological Sciences, 363(1490), 321–339. 10.1098/rstb.2007.2142 PMC2606753 17638690

[ece39178-bib-0030] Moshkin, M. P. , Dobrotvorsky, A. K. , Mak, V. V. , Panov, V. V. , & Dobrotvorskaya, E. A. (1998). Variability of immune response to heterologous erythrocytes during population cycles of red (*Clethrionomys rutilus*) and bank (*Cl. glareolus*) voles. Oikos, 82, 131–138.

[ece39178-bib-0031] Nelson, R. J. (2004). Seasonal immune function and sickness responses. Trends in Immunology, 25, 187–192. 10.1016/j.it.2004.02.001 15039045

[ece39178-bib-0032] Nelson, R. J. , & Demas, G. E. (1996). Seasonal changes in immune function. The Quarterly Review of Biology, 71(4), 511–548. 8987173 10.1086/419555

[ece39178-bib-0033] Novikov, E. A. , Mak, V. V. , Panov, V. V. , & Moshkin, M. P. (2010). Humoral immune response to non‐replicating antigens and infection with taiga tick (*Ixodes persulcatus*, Acarina, Ixodidae) of northern red‐backed voles (*Clethrionomys rutilus*, Rodentia, Cricetidae). Zoologicheskii Zhurnal, 89, 106–114.

[ece39178-bib-0034] Olenev, G. V. (2002). Alternative types of ontogeny in cyclomorphic rodents and their role in population dynamics: An ecological analysis. Russian Journal of Ecology, 33(5), 321–331.

[ece39178-bib-0035] Ots, I. , Kerminov, A. , Ivankina, E. V. , Ilyina, T. A. , & Horak, P. (2001). Immune challenge affects basal metabolic activity in wintering great tits. Proceedings of the Royal Society B: Biological Sciences, 268, 1175–1181. 10.1098/rspb.2001.1636 PMC1088724 11375106

[ece39178-bib-0036] Paynter, S. , Sly, P. D. , Ware, R. S. , Williams, G. , & Weinstein, P. (2014). The importance of the local environment in the transmission of respiratory syncytial virus. Science of the Total Environment, 493, 521–525. 24973721 10.1016/j.scitotenv.2014.06.021

[ece39178-bib-0037] Rogovin, K. A. , & Naidenko, S. V. (2010). Noninvasive assessment of stress in bank voles (*Myodes glareolus*, Cricetidae, Rodentia) by means of enzyme linked immunosorbent assay (ELISA). Biology Bulletin, 37(9), 959–964.

[ece39178-bib-0038] Roth, T. , Sammak, R. , & Foley, J. (2018). Prevalence and seasonality of fleas associated with california ground squirrels and the potential risk of tularemia in an outdoor non‐human primate research facility. Journal of Medical Entomology, 55(2), 452–458. 29202201 10.1093/jme/tjx201

[ece39178-bib-0039] Safronov, V. M. (2009). Adaptive specific features of thermal regulation and maintaining energy balance in mouse‐like rodents. Biology, 4(8), 47–61.

[ece39178-bib-0040] Saitoh, T. , Stenseth, N. C. , Viljugrein, H. , & Kittilsen, M. O. (2003). Mechanisms of density dependence in fluctuating vole populations: Deducing annual density dependence from seasonal processes. Population Ecology, 45, 165–173.

[ece39178-bib-0041] Shudo, E. , & Iwasa, Y. (2001). Inducible defense against pathogens and parasites: Optimal choice among multiple options. Journal of Theoretical Biology, 209(2), 233–247. 11401465 10.1006/jtbi.2000.2259

[ece39178-bib-0042] Siegel, S. , & Castellan, N. J. (1988). Nonparametric Statistics for the Behavioral Sciences (pp. 213–214). McGraw‐Hill.

[ece39178-bib-0043] Sinclair, J. A. , & Lochmiller, R. L. (2000). The winter immunoenhancement hypothesis: Associations among immunity, density, and survival in prairie vole (*Microtus ochrogaster*) populations. Canadian Journal of Zoology, 78, 254–264. 10.1139/cjz-78-2-254

[ece39178-bib-0044] Soininen, E. M. , Ravolainen, V. T. , Brathen, K. A. , Yoccoz, N. G. , Gielly, L. , & Rolf, A. I. (2013). Arctic small rodents have diverse diets and flexible food selection. PLoS One, 8(6), e68128. 10.1371/journal.pone.0068128 23826371 PMC3694920

[ece39178-bib-0045] Vasilieva, N. Y. , Khrushchova, A. M. , Kuptsov, A. V. , Shekarova, O. N. , Sokolova, O. V. , Wang, D. , & Rogovin, K. A. (2020). On the winter enhancement of adaptive humoral immunity: Hypothesis testing in desert hamsters (*Phodopus roborovskii*: Cricetidae, Rodentia) kept under long‐day and short‐day photoperiod. Integrative Zoology, 15(3), 232–247. 31773894 10.1111/1749-4877.12419

[ece39178-bib-0046] Xu, D.‐L. , & Hu, X.‐K. (2017). Photoperiod and temperature differently affect immune function in striped hamsters (*Cricetulus barabensis*). Comparative Biochemistry and Physiology – Part A: Molecular and Integrative Physiology, 204, 211–218. 10.1016/j.cbpa.2016.12.009 27956167

[ece39178-bib-0047] Zavjalov, E. L. , Gerlinskaya, L. A. , & Evsikov, V. I. (2003). Assessment of the stress of the bank vole, *Clethrionomys glareolus* (Rodentidae, Rodentia), by the level of corticosterone in faeces. Zoologicheskii Zhurnal, 82(4), 508–513.

[ece39178-bib-0048] Zejda, J. (1971). Differential growth of three cohorts of the bank vole, *Clethrionomys glareolus*, Schreb., 1780. Zoologicke Listy, 20, 229–245.

[ece39178-bib-0049] Zuk, M. , & Stoehr, A. M. (2002). Immune defense and host life history. American Naturalist, 160(4), 9–22. 10.1086/342131 18707455

